# The Use of Electronic Data Capture Tools in Clinical Trials: Web-Survey of 259 Canadian Trials

**DOI:** 10.2196/jmir.1120

**Published:** 2009-03-09

**Authors:** Khaled El Emam, Elizabeth Jonker, Margaret Sampson, Karmela Krleža-Jerić, Angelica Neisa

**Affiliations:** ^3^Canadian Institutes of Health ResearchOttawaONCanada; ^2^PediatricsFaculty of MedicineUniversity of OttawaOttawaONCanada; ^1^Children’s Hospital of Eastern Ontario Research InstituteOttawaONCanada

**Keywords:** Clinical trials, diffusion of innovation, electronic data capture, data collection

## Abstract

**Background:**

Electronic data capture (EDC) tools provide automated support for data collection, reporting, query resolution, randomization, and validation, among other features, for clinical trials. There is a trend toward greater adoption of EDC tools in clinical trials, but there is also uncertainty about how many trials are actually using this technology in practice. A systematic review of EDC adoption surveys conducted up to 2007 concluded that only 20% of trials are using EDC systems, but previous surveys had weaknesses.

**Objectives:**

Our primary objective was to estimate the proportion of phase II/III/IV Canadian clinical trials that used an EDC system in 2006 and 2007. The secondary objectives were to investigate the factors that can have an impact on adoption and to develop a scale to assess the extent of sophistication of EDC systems.

**Methods:**

We conducted a Web survey to estimate the proportion of trials that were using an EDC system. The survey was sent to the Canadian site coordinators for 331 trials. We also developed and validated a scale using Guttman scaling to assess the extent of sophistication of EDC systems. Trials using EDC were compared by the level of sophistication of their systems.

**Results:**

We had a 78.2% response rate (259/331) for the survey. It is estimated that 41% (95% CI 37.5%-44%) of clinical trials were using an EDC system. Trials funded by academic institutions, government, and foundations were less likely to use an EDC system compared to those sponsored by industry. Also, larger trials tended to be more likely to adopt EDC. The EDC sophistication scale had six levels and a coefficient of reproducibility of 0.901 (*P*< .001) and a coefficient of scalability of 0.79. There was no difference in sophistication based on the funding source, but pediatric trials were likely to use a more sophisticated EDC system.

**Conclusion:**

The adoption of EDC systems in clinical trials in Canada is higher than the literature indicated: a large proportion of clinical trials in Canada use some form of automated data capture system. To inform future adoption, research should gather stronger evidence on the costs and benefits of using different EDC systems.

## Introduction

Electronic data capture (EDC) systems are used in all phases of clinical trials to collect, manage, and report clinical and laboratory data [1]. The capabilities of those systems vary from the basic stand-alone database used for data entry in a single-site trial, to the more sophisticated systems supporting multisite international trials with remote data entry over the Web, data validation at the time of entry (eg, checking for out-of-range values or impossible combinations of values), real-time status reporting overall and per site, participant status tracking, and on-demand subject randomization.

Such systems have been discussed in the literature for more than a decade [2,3]. There are a handful of studies suggesting that the use of EDC systems can accelerate clinical trial start-up, reduce the overall duration of a trial, and reduce data errors [4-6]. To the extent that these positive results can be generalized, they make the case for wider adoption of this technology in clinical trials.

The number of published trials that use an EDC system has been rising [7], and there have been claims of a rapid uptake of this technology in clinical trials [8,9]. However, this optimistic assessment is inconsistent with reports that the failure rate of EDC adoption is as high as 70% [10], and, notwithstanding methodological weakness in the existing evidence, only 20% of trials are using EDC systems (see the systematic review in Multimedia Appendix 1). If indeed the failure rate is so high and the adoption rate is somewhat low, then either the technology is not quite ready for use or there are extreme difficulties being experienced in changing the practice of clinical trials to accommodate more automation. Should that be the case, then future research should investigate the quality and sophistication of EDC solutions and address the change management issues in the adoption of such a new technology in clinical trials workflows.

The primary objective of this study was to estimate the proportion of phase II/III/IV Canadian clinical trials that used an EDC system in 2006 and 2007. The secondary objective was to investigate three factors that can have an impact on adoption: trial size, source of funding, and type of participants.

Trial size was measured in terms of the target number of patients recruited and number of sites. We expected that larger trials would be more likely to use an EDC system. The total cost of a trial is partially driven by the number of patients recruited. Therefore, if a technology reduces the effort spent per patient (eg, on date entry and query resolution), then larger trials will likely benefit more from EDC technology than smaller trials, making it more likely that EDC would be adopted in the larger trials.

Source of funding indicated whether the trial was commercially or academically/foundation funded. Controlling for size differences, we expected commercially sponsored trials to be more likely to use an EDC system. A main reason is that academic/foundation trials are less likely to have the funding to license and implement an enterprise-level computerized system.

Type of participant indicates whether the participants were adult or pediatric. We had no a priori expectations about the direction of impact of this factor and included it for exploratory purposes.

The contributions of this work are as follows: (1) We have developed a scale to assess whether an EDC system is being used and determine its level of sophistication, (2) We have performed a content validation and unidimensional (Guttman) scaling of the EDC sophistication scale, (3) We provided an updated estimate of EDC adoption in Canadian clinical trials, and (4) We have identified which trial factors have an impact on EDC adoption.

## Methods

### Measurement

#### Definition of an EDC System

Previous studies of EDC adoption did not have a clear definition of what precisely an EDC system is (see the review in Multimedia Appendix 1). This increases the risk of variation among survey respondents’ interpretation of the meaning of an EDC system and consequently increases the potential for error in the survey results.

The use of an EDC system in a clinical trial does not preclude the parallel use of paper case report forms (CRFs). Because of uncertainty about whether regulatory authorities will accept electronic documents as source documents (e-source), many sites still maintain source documents on paper [11-13]. With an EDC system in use, these data are typed into an electronic system by the site personnel for submission to the central database. There are also studies where data are being collected from/by different types of individuals using multiple modes of data entry. For example, nurses may enter data into an electronic system, but patient diaries are on paper, or vice versa. Therefore, in practice, paper and electronic systems coexist at the trial sites.

To ensure consistent interpretation of what an EDC system is in our study, we asked questions about the features of the systems that were used in the clinical trial. If an electronic system was used for data capture and management and it had at least a minimum set of features, then it was considered to be an EDC system. We define a minimum set of features as allowing trial sites to submit data electronically into the central database and to be able to query that central database for reports and aggregate statistics.

All trials have to enter/transfer their data at some point into an electronic database or file for analysis. If trial sites send paper CRFs or fax them to a central coordinating site and the on-paper data are transcribed into a central database, that database would not be considered an EDC system by our definition because data are not submitted electronically.

The feature set we used was obtained from comparative product reviews [7,14] and Food and Drug Administration (FDA) regulations, namely the FDA’s 21 CFR Part 11 regulation “Electronic Records; Electronic Signatures” [15-19], which regulates the use of EDC in trials. A content validation study was performed to ensure that we had adequate coverage of critical EDC system features that are used in practice. The questionnaire development process, pilot testing, and the final questionnaire are provided in Multimedia Appendix 2.

#### The EDC Sophistication Scale

We can divide EDC systems into those offering “basic” and those offering “advanced” features. Thus, it is natural to have variation in the features that are implemented in different EDC systems. The more features that an EDC system implements, the more “advanced” it is considered.

If an EDC system implements the “advanced” features, then it would by definition also implement the “basic” features as well. The former would include the latter. This type of cumulative relationship can be modeled through a Guttman scalogram [20,21].

The original intention of Guttman scaling was that such a scale would measure a single underlying dimension of a phenomenon (eg, job satisfaction or symptoms of fear during battle [22]). The basic thesis of Guttman scaling is that it is possible to determine which items were endorsed by a subject from the knowledge of their total score (ie, an unweighted sum of their responses). Assume that we have a five-item scale. Then, in a Guttman scale, all subjects who endorse four items do so with respect to the *same* four items; those who endorse three items do so with respect to the *same* three items. Furthermore, these three items are among the four items endorsed by those who endorse four items.

Previous applications of the Guttman scaling approach include the study of the evolution, progression, or growth of various objects. For example, anthropologists utilize scalogram techniques for studying the evolution of cultures [23], and sociologists, in the study of the evolution of legal institutions [24].

The Guttman scale is suitable for defining cumulative functionality levels for an EDC system such that if a system implements, say, feature 5, then it is likely to have also implemented features 1, 2, 3, and 4. If features can be ordered, then the higher features signify more EDC sophistication.

We therefore used Guttman scalogram analysis to create an ordered scale of EDC sophistication, with lower scores indicating an EDC system that is more basic with fewer features, and higher scores indicating an EDC system that is more advanced. The coefficient of reproducibility [25,26] and the coefficient of scalability [27] are used to evaluate how well the data fit the cumulative scale. Common acceptable thresholds for these two indices are 0.9 for reproducibility and 0.6 for scalability [28].

### Sampling Frame

Clinical trials with Canadian sites were identified through two main international clinical trials registries: ClinicalTrials.gov and Current Controlled Trials. Such registries have been used in the past to perform descriptive analysis, such as on the global growth of clinical trials [29]. Not all of the entries in these registries are, strictly speaking, controlled trials since they include phase IV observational studies as well.

Since the 1997 FDA Modernization Act, FDA-regulated efficacy drug trials for serious or life-threatening diseases or conditions have to be registered with ClinicalTrials.gov [30]. One analysis conducted in 2003 noted that there were more than 2000 investigational new drugs and 731 nongovernment-sponsored trials registered (around 37% registration rate) [31]. The 2007 FDA Amendments Act considerably expanded the scope of trials to be registered by including all trials except phase I and imposed penalties for noncompliance. Following the registration requirement by the major medical journals, led by the International Committee of Medical Journal Editors in 2005 [32,33], registrations with ClinicalTrials.gov have increased dramatically [34]. We therefore expected that ClinicalTrials.gov would have good coverage of commercial clinical trials, including those with Canadian sponsors as well as non-Canadian sponsors with sites in Canada.

The Canadian Institutes of Health Research (CIHR), which is the main public funding agency for health research in Canada, has a requirement that the randomized controlled trials it funds be registered with an International Standard Randomised Controlled Trial Number (ISRCTN) and that basic information about each trial be posted on the ISRCTN registry (Current Controlled Trials) [35]. We therefore expected that between the ISRCTN registry and ClinicalTrials.gov, most of the Canadian non-commercial trials would be captured.

Our sampling frame consists of registered trials that were running in Canada from January 1, 2006, to December 31, 2007 inclusive. This means trials were included that were started or terminated during that period, as well as ongoing trials that started before 2006 and those that were still running at the end of 2007.

### Sample Size

Based on our systematic review (see Multimedia Appendix 1), we expected that 20% of all trials would be using an EDC system. For an estimate of the proportion of trials using EDC with a 95% confidence interval ± 5%, we would need 246 observations. It is reasonable to expect a 40% unit response rate for a Web-based survey [36,37]. Therefore, we needed to survey at least 615 clinical trials.

To analyze the factors affecting adoption, we constructed a logistic regression model [38] with a binary outcome (EDC adoption) of the form *Adoption* ~ *Type F* + *Type P* + log (*Size*), where *Type F* was a dummy variable indicating whether the trial was academic or industry, *Type P* was a dummy variable indicating whether the trial participants were adult or pediatric, and *Size* was the target patient recruitment.

Funding source and size were available/discernable from the two trial registries. We performed a log transformation on the target patient recruitment variable to ameliorate the heavy tail (the transformed variable does not deviate from normality according to the Kolmogorov-Smirnov test).

For the impact of size and whether a trial was academic or industry, our initial hypotheses in the introduction were directional. Therefore, we used one-tailed tests on the parameters for these two variables in our logistic model. For the adult versus pediatric impact on adoption, our initial hypothesis was nondirectional. Therefore, we adopted a two-tailed test for that analysis.

At 80% power and a baseline adoption probability of 0.2, a 246 sample size for the multivariate logistic regression model can detect an odds ratio (OR) of 1.57 at a one-tailed alpha level of 0.05 for a one standard deviation increase in the log target recruitment variable [39], which represents a plausible increase in the probability of adoption. Similarly, the OR for the binary academic/industry detectable at the same sample size is 2.26 for a change from academia to industry for a one-tailed alpha level of 0.05, and 3.7 for pediatric to adult for a two-tailed alpha level of 0.05. Therefore, the impact of type of participant would have to be quite large to be detectable.

### Approach

The commercial SurveyMonkey system was used to run and manage the survey.

It has been noted that contact information in online clinical trials registries has created a burden on principal investigators (PIs) through excessive emails from patients, other clinicians, and direct marketers [40,41]. Therefore, we expected that most PIs who are the main contacts in these registries would be unlikely to respond to the survey themselves. Keeping that in mind, and considering that coordinators are the end users of an EDC system and would have the operational experience of an EDC if it was used in a trial, and that they would be more likely to respond to the questionnaire, we decided to survey Canadian site coordinators.

The registries did not always provide detailed contact information for the site coordinators. In such cases, we had to determine the contact information for the Canadian site coordinators ourselves. Two approaches were followed. Initially, an email was sent to the main contact of the clinical trial listed with ClinicalTrials.gov or Current Controlled Trials asking him or her to send us the contact information for the Canadian sites. If the above did not work (eg, often trials do not have contact information if the trial has stopped recruiting, the trial may provide a generic sponsor address as a contact, or a PI contact may not respond), we contacted the administrative person responsible for clinical research at the sponsor or for the Canadian sites listed in the registries asking for assistance in locating the coordinator.

### Administration

Each study coordinator was contacted by email inviting him or her to participate in the survey. Three reminders were sent out at one-week intervals. Respondents were also entered into a raffle for three iPod Shuffles. A summary of the Web survey details according to the CHERRIES guidelines [42] is provided in Multimedia Appendix 3.

### Analysis

The adoption rates are presented descriptively as a proportion with 95% confidence intervals [43].

The overall logistic regression model significance test is performed using the G statistic [38], and goodness of fit is evaluated using the Nagelkerke pseudo-*R* ^2^ [44,45]. This pseudo-*R* ^2^ tends to have low values compared to what one would expect in ordinary least squares regression models. Collinearity among the independent variables was assessed using the condition number [46,47]. In general, a condition number above 30 is considered problematic. Influential observations were detected using the delta-beta coefficient [48] and investigated.

## Results

### Description of Trials

In total, there were 947 registered trials with sites in Canada that were running at some point in time during 2006 and 2007. This excludes five trials for which the central coordinating site was our home institution.

The median target number of participants to recruit was 226; the median number of sites was 5, and the median percentage of sites that were Canadian was 100%. The number of patients and sites are skewed, with some trials having a much larger recruitment target: the largest trial had 782 sites and a target recruitment of 35,000 participants. There were 498/947 trials (52.6%) funded by academic institutions, government funding agencies, or foundations (henceforth “academic” trials), and the remaining 449/947 trials (47.4%) were funded by industry (henceforth “industry” trials). Therefore, there was a relatively equal split of trials in terms of funding source.

As can be seen in Table 1, industry trials tended to be approximately three times larger in terms of participant recruitment, with substantially more overall sites but proportionally fewer that were Canadian. There were large multicenter academic studies, with the largest academic study having 782 sites of which 14 were in Canada, and the largest industry trial having 757 sites of which 29 were Canadian.

**Table 1:** Differences between academic and industry trials (two-tailed tests)

	Academic (median)	Industry (median)	*P* value (Mann-Whitney U Test [49])
Number of participants	130	400	< .001
Total sites	1	39	< .001
Canadian sites	100%	11%	< .001

There were 84/947 pediatric-only trials (approximately 9%), and 863/947 adult trials (approximately 91%). In this classification, trials that included adults and youth in their recruitment criteria were classified as adult since they did not focus specifically on a pediatric population. Adult trials were equally likely to be academic as industry (433 vs 430), whereas pediatric trials were much more likely to be academic (chi-square test: *P*< .001).

As can be seen in Table 2, adult trials tended to be almost one and a half times as large as pediatric trials in terms of participant recruitment, with more overall sites but proportionally fewer that were Canadian.

**Table 2:** Differences between adult and pediatric trials (two-tailed tests)

	Adult (median)	Pediatric (median)	*P* value (Mann-Whitney U Test [49])
Number of participants	236	141	< .001
Total sites	6	1	.003
Canadian sites	57%	100%	.001

### Response Rate and Nonresponse

**Figure 1:**
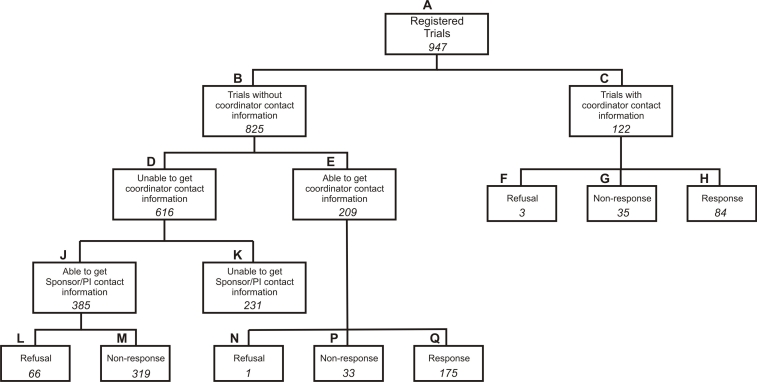
Responses to the survey

As shown in Figure 1, we were able to get study contact information for 716/947 trials (75.6% of the total). These are nodes C, E, and J. C and E represent the 331 trials for which we were able to obtain Canadian site coordinator contact information and that were sent the actual survey. These represent 46.2% of contactable trials (331/716).

Trials for which we did not get contact information tended to be larger industry trials. For some, no contact information was available at all. For others, we had a sponsor or PI contact, whom we followed up with to get Canadian site coordinator contact information. In Figure 1, D represents the trials for which there was insufficient contact information in the registry; for these trials, we tried to get coordinator contact information by contacting the sponsor or PI (J), or it was not possible to get sponsor or PI contact information (K).

Reasons given by sponsors or PIs for refusing to provide contact information (node L in Figure 1) included the following: (1) there was a need to get coordinator consent or local site Research Ethics Board approval first before giving us the information because the coordinators would be participating in a research study, (2) the federal privacy legislation (PIPEDA) bars the disclosure of the coordinators’ business contact information or that this was confidential (proprietary) information, and (3) the site coordinators do not have time to participate in a survey or the contact does not have time to provide us with the coordinators’ contact information.

In all of our subsequent analyses, weights were used to ensure that our responding sample adequately represented the population of Canadian trials [50].

Out of the 331 trials for which we obtained coordinator contacts, 72 did not respond (78% response rate to the survey). We compared those nonrespondents to respondents on the same set of variables. There was no statistically significant difference in the response rates for industry and for academic trials by chi-square criteria. Neither was there a statistically significant difference in response rate for adult trials and for pediatric trials. Furthermore, we did not find any significant differences between survey respondents and nonrespondents on the other three variables (number of patients, number of sites, and proportion of Canadian sites) at a Bonferroni adjusted alpha level of 0.05.

Of the 331 trial coordinators to whom we sent the survey, we wanted to determine if there was a nonresponse bias in terms of their adoption of EDC. A common way to evaluate this is to compare early versus late respondents, where late respondents are a proxy for nonrespondents [51]. We found no significant difference by chi-square criteria.

### EDC Adoption Rate

Trials that did not select any of the features were clearly not EDC system users. There was considerable variation in the features of the electronic systems that the remaining trials used. System features can be grouped into six cumulative levels of sophistication (see Table 3): (1) f1, (2) f2 to f4, (3) f5 and f6, (4) f7, (5) f8, and (6) f9. The grouping of features at levels 2 and 3 is done because these features almost always occurred together in EDC systems used by our respondents. We performed a Guttman scaling on the six levels of EDC sophistication. The coefficient of reproducibility for the Guttman scale is 0.901 (*P*< .001), and the coefficient of scalability is 0.79. Such high coefficients provide evidence that the features in EDC systems are cumulative according to our six levels, and therefore the level can be used as a unidimensional score of EDC sophistication.

Based on our definition, systems at a sophistication level of 1 would not be considered an EDC system. For example, if a coordinating center used a password-protected stand-alone database to manually enter paper CRFs that were sent in by courier from other sites, then it would have a system at the first level of sophistication.

Therefore, we only considered systems with a sophistication level of 2 and above as an EDC system. It is estimated that 41% of all trials (95% CI 37.5%-44%) are using an EDC system with a sophistication level of 2 or above.

**Table 3:** The grouping of features into a six-level cumulative scale of EDC sophistication as determined through a Guttman scalogram analysis: higher levels signify more sophistication

Sophistication Level	Features	
1	f1.	There is a unique account and password for each user to access the online system.
2	f2.	Subject visit data are entered by sites through a Web interface into electronic case report forms (eCRFs).
	f3.	The completion status of each eCRF for each subject can be tracked automatically online; for example, you can see which visits have complete data and which still have incomplete eCRFs for each subject.
	f4.	The system provides an audit trail for all data entry and data modification.
3	f5.	Data validation happens automatically when data are entered into the eCRF (either right away or when the user presses the SUBMIT button), for example, to check for out-of-range values.
	f6.	The system will automatically log the user off after a period of inactivity.
4	f7.	Subjects are randomized automatically, either through an automated telephone response system or through a Web interface.
5	f8.	Subject recruitment can be tracked online for each site; for example, the user can see a graph of recruited and not withdrawn subjects over time.
6	f9.	The system allows tracking of medication inventory at the sites.

The most basic EDC systems in use today have Web-based data entry forms, form completion tracking, and audit trails. Automated randomization is a feature of relatively sophisticated EDC systems. Few trials are able to track subject recruitment over time, and tracking medication inventory is quite uncommon. The median EDC sophistication level was 4 for both academic and industry trials. The median EDC sophistication level for adult trials was 4, and for pediatric trials it was 5. This difference was statistically significant (Mann-Whitney U two-tailed test, *P*= .003).

The logistic regression model to predict EDC adoption had a Nagelkerke *R* ^2^ of 0.22. The dummy variable indicating whether the trial was industry or academic was statistically significant (OR = 1.52; one-tailed *P*= .002), and the size variable was also statistically significant (OR = 1.44; one-tailed *P*< .001). Whether a trial had pediatric or adult participants was not significant using a two-tailed test. This suggests that larger trials tended to be more likely to adopt EDC and that industry trials were also more likely to adopt EDC. Whether the trial was adult or pediatric did not make a difference in the adoption of EDC.

## Discussion

### Summary

The clinical trials landscape in Canada is evenly split between academic and industry trials. However, industry trials tended to be larger with more patients and sites. More than 90% of trials were of adults, and these tended to be larger than pediatric trials. Our results reveal that the 41% adoption rate of EDC systems in Canadian clinical trials is twice the commonly cited value. Larger trials and those sponsored by industry are more likely to use an EDC system. We found that the type of participants did not have an impact on adoption, but this may be because the sample was under-powered to detect this effect given that the distribution of adult/pediatric trials was quite skewed.

While there is no difference in the level of sophistication of EDC systems used between academic and industry trials, pediatric trials tended to have more sophisticated EDC use than those with predominantly adult participants.

It is not surprising that industry-funded trials included in the sample were larger than academic ones. Pharmaceutical companies in Canada invested between $1.1425 billion and $1.67 billion on R&D in 2003 [52,53], of which between $487.5 million and $668 million was on clinical trials [53,54]. These numbers exclude stakeholders such as the biotechnology industry [55] and therefore are expected to be an underestimate. In comparison, the main academic health research funding body in Canada, CIHR, spent only $57 million on clinical trials research during the same period.

To the extent that the need for heavy investments in information technology (IT) can act as a barrier to use, cost would have been a deterrent for academically funded trials to use IT to the same extent as industry trials during the 2006-2007 period that we studied. This concurs with the observation that the median number of sites for academic trials was one; it may be more difficult to justify an investment in EDC for single-site trials. However, recently more EDC systems are adopting the Software as a Service (SaaS) model, where sites access the EDC through their Web browser. Such systems demand less of an IT capacity at each site to get started and do not require a large capital expenditure at the outset of the study to purchase equipment and software licences. Therefore, over time it is plausible that the adoption rate for academic trials will catch up to industry trials.

Despite academic trials having a lower adoption rate, there were no differences in terms of the sophistication of the EDC systems that were used by industry-sponsored and academic trials. Therefore, when academic trials do adopt an EDC system, they do not opt for systems with fewer features.

### Practical Implications

**Figure 2:**
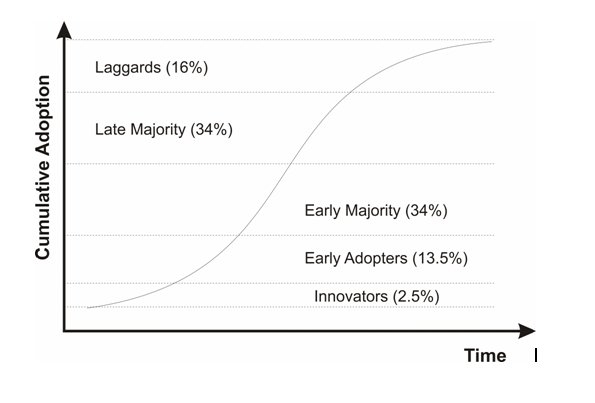
The S-shaped diffusion of technology curve

A commonly accepted descriptive model of the diffusion of innovations is an S-shaped curve, as shown in Figure 2 [56], which characterizes many technological innovations, irrespective of the technology. For example, one study reviewed the adoption patterns of a variety of 20th century consumer products (eg, washing machines, videocassette recorders) and found that they follow the same adoption curve [57], while Teng and Grover developed historical diffusion curves for general information technologies (eg, personal computers, email) [58]. Health care information technologies, including electronic health records, order entry systems, and mobile devices, have also been examined within this diffusion framework [59-63].

To the extent that this model applies to EDC adoption, we are currently in the steepest point of adoption among the early majority of Canadian trials. Consequently, it would be reasonable to expect increased use of EDC systems in trials in the immediate future. This trend is consistent with other evidence showing rising adoption of health IT in general, and specifically, electronic health records [60,64-70], in medical centers and practices.

High adoption rates of EDC systems have a number of practical and research implications. First, the characteristics of the adopters change over time and so does the nature of suitable evidence to inform their adoption decisions [56]. For example, innovators (the first 2.5% who adopt a new technology) do not need evidence to make an adoption decision. Early adopters (the next 13.5%) are satisfied with case studies and examples of successful adoption and benefits. The early majority require stronger evidence of benefits. There is therefore a strong requirement for more systematic evaluation of EDC systems to quantify the costs and benefits of their use and the contexts in which benefits do or do not materialize in order to address the information needs of the early majority.

Second, EDC systems make it much more practical to make the frequent design changes that are required in adaptive clinical trials [71]. One would therefore expect to see a parallel rise in the use of adaptive trial designs.

Third, for commercial trials, electronic submissions to regulatory authorities would become more practical with the increased use of EDC systems.

Finally, to the extent that EDC improves the data quality and efficiency of trials, higher EDC adoption would be expected to enable such benefits to materialize in the future.

In terms of the EDC systems themselves, the median sophistication level of EDC systems indicates that many trials are not able to track recruitment in real time. This suggests an important feature that EDC developers need to make sure is added to their systems.

### Comparison to Previous Work

Our systematic review of the literature (see Multimedia Appendix 1) indicated that only 20% of clinical trials were using an EDC system. It is useful to explore why this number is very different from our results. This large discrepancy can be explained in five possible ways. First, the 20% adoption was true a few years ago and adoption has progressed significantly over the intervening period, reaching the levels we have reported here for 2006-2007. Second, the studies providing the 20% adoption numbers were methodologically weak and therefore this number is unreliable. Third, previous studies used a different unit of analysis—many were reporting on the proportion of pharmaceutical companies and contract research organizations (CROs) that were using EDC rather than the proportion of trials. However, the unit of analysis was often not easily discernable from the published accounts. Fourth, previous studies were not specific to clinical trials in Canada, as opposed to our current results. Finally, previous work did not have a consistent and precise definition of what an EDC system is, and this may have contributed to different surveys not measuring the same thing and classifying systems as EDC differently than us.

It is most likely that reality is a mixture of the above five reasons.

### Future Work

It would be of value to track the adoption of EDC over time using regular surveys similar to the current one. This will provide evidence as to whether the adoption is actually following the S-shaped adoption curve in Figure 2 as we have postulated.

Additional comparisons with the United States and Europe would be informative. If there are significant regional differences in adoption rates, then there may be policy or structural choices that explain the differential. For example, if one region has adopted a certain set of policies or incentives, or has an existing health informatics infrastructure that supports the use of EDC, then other regions may consider duplicating those drivers to accelerate their EDC adoption rates.

There are other factors that could have an impact on the adoption of EDC that would be useful to investigate in future research. For example, for academically funded trials, one would consider the age of the PI, his or her technical skill/knowledge, the existence of a senior informatics person to provide support, whether there is an existing research systems infrastructure in place with programming or database resources available for investigators to use, and whether or not the academic institution already has a sophisticated EDC system available for use by any investigators. For industry-funded trials, one could consider the size of the organization running the trial (whether it is the industry sponsor or a CRO), the size of trials usually conducted, and the number of trials conducted per year in the geographical region of study (say, Canada or the United States).

Since we have developed an EDC sophistication measure, it would now be easier to evaluate the relationship between EDC sophistication and the benefits of EDC. One can hypothesize that more sophisticated EDC use will be associated with greater benefits, such as faster trial completion and fewer data errors.

### Limitations

One limitation of our results is that individuals conducting clinical trials may not have registered their trials [40], suggesting that some investigator-initiated trials may not be in the registries. If that is indeed the case for trials with sites in Canada, then unregistered trials may introduce a bias if they differ systematically in terms of their adoption of EDC technology and/or size.

Our results are limited to Canada, and the adoption rates may be different in other jurisdictions.

## Multimedia Appendix 1

Systematic Review of EDC Adoption Surveys

## Multimedia Appendix 2

Questionnaire Development and Validation

## Multimedia Appendix 3

CHERRIES Summary

## Abbreviations

CIHR: Canadian Institutes of Health Research
CRF: case report form
CRO: contract research organization
eCRF: electronic case report form
EDC: electronic data capture
FDA: Food and Drug Administration
ISRCTN: International Standard Randomised Controlled Trial Number
IT: information technology
PI: principal investigator
