# Supplementary material for: The Use of Electronic Data Capture Tools in Clinical Trials: Web-Survey of 259 Canadian Trials
Source: J Med Internet Res. 2009 Mar 9;11(1):e8. doi: 10.2196/jmir.1120 (PMC2762772; doi:10.2196/jmir.1120)
Supplement: Supplementary file 1 [file jmir_v11i1e8_app1.pdf]

Multimedia Appendix 1: Systematic Review of EDC Adoption Surveys

We performed a systematic review of surveys of EDC adoption to determine what is known about EDC adoption trends. Performing a systematic review of surveys to gauge the extent of adoption of information technology in healthcare settings is a common approach. For example, systematic reviews of surveys were used to evaluate the adoption of personal digital assistants among providers [1] and assess the use and adoption of electronic health records in the US [2]. We therefore follow a similar approach.

Search Strategy

Searches were developed for MEDLINE(R) (1996 to October Week 4 2006), Cochrane Methodology Register (Cochrane Library, Issue 4, 2006), Scopus and Google Scholar (both as of November 15, 2006). In addition, four relevant references were selected to use as seeds for the PubMed Related Article search. Related articles were limited to the last 10 years. Searches were limited to English language articles. The search strategies are listed in Figure 1, as are the PubMed related article seeds.

Additional efforts to locate relevant studies included contacting experts in the field, as well as conducting Internet searches of pertinent organizations' websites. Through these methods, the reviewers nominated eight potentially relevant articles.

At the completion of the systematic review an update search of MEDLINE(R) until week 1 of October 2007 was performed and relevant articles were identified and screened for inclusion.

MEDLINE search

Ovid MEDLINE(R) <1996 to October Week 4 2006>

1 exp Microcomputers/ 3350
2 computer$.tw. 66523
3 (internet or web or online or tablet or pen based).tw. 30602
4 (pda$ or personal digital assistant$ or handheld or hand held).tw. 4398
5 (palm adj (top or pilot)).tw. 49
6 laptop$.tw. 275
7 (tablet$ or pen based or wireless).tw. 10540
8 electronic$.tw. 20981
9 exp computing methodologies/ 224209
10 or/1-9 298794
11 exp Data collection/ 527179
12 Database Management Systems/ 3139
13 adverse drug reaction reporting systems/ 2447
14 Automatic data processing/ 2785
15 case report form$.tw. 169
16 crf$.tw. 4187
17 (diary or diaries).tw. 5123
18 (electronic adj3 outcome$).tw. 39
19 e-PRO.tw. 6
20 (data adj2 capture).tw. 380
21 data collection.tw. 12966
22 or/11-21 549410
23 trialmaster.tw. 1
24 trialstat.tw. 0
25 webipa$.tw. 1
26 (afferenz or megasoft).tw. 0
27 clickfind.tw. 0
28 clinsource.tw. 0
29 datalabs$.tw. 0
30 datatrak$.tw. 0
31 datatrial.tw. 0
32 document solutions group$.tw. 0
33 DSG.tw. 338
34 DZS software solutions$.tw. 0
35 eresearch technolog$.tw. 0
36 etrials worldwide$.tw. 0
37 (ICTI or iTrial).tw. 3
38 infermed$.tw. 2
39 labware.tw. 14
40 logos technolog$.tw. 0
41 medidata solution$.tw. 0
42 nextrial$.tw. 0

43 ninaza.tw. 0
44 omnicomm system$.tw. 1
45 oracle corpor$.tw. 4
46 phase forward$.tw. 1
47 phoenix data system$.tw. 0
48 acumen healthcare.tw. 0
49 advanced clinical systems.tw. 1
50 avalis$.tw. 0
51 clarix.tw. 0
52 compleware.tw. 0
53 data capture internation$.tw. 0
54 kika.tw. 6
55 lifetree technology$.tw. 0
56 maaguzi.tw. 0
57 (new england research institute or NERI).tw. 11
58 pharmavigilant.tw. 0
59 (phosco or guillemot design$).tw. 0
60 studybuilder$.tw. 0
61 electronic data capture.tw. 16
62 edc.tw. 502
63 (clinical data management system$ or cdms).tw. 67
64 clinical trial management software.tw. 0
65 or/23-64 961
66 65 or (10 and 22) 40541
67 exp Clinical Trials/ 89856
68 66 and 67 1774
69 (ut or td).fs. 136163
70 (rate$ or adopt$ or uptake or up-take or trend$).tw. 683767
71 forecasting/ 30771
72 or/69-71 807087
73 68 and 72 611
74 limit 73 to (english and yr="1997 - 2007") 578

Cochrane Methodology Register search

Cochrane Library, Issue4, 2006

#1 MeSH descriptor Microcomputers explode all trees 119
#2 computer*:kw 5232
#3 (internet OR web OR online OR tablet OR pen based):kw 3096
#4 (PDA* OR personal digital assistant* OR handheld OR hand held):kw 25
#5 palm NEXT (top OR pilot):kw 0
#6 laptop*:kw 0
#7 (tablet* OR pen based OR wireless):kw 2574
#8 electronic*:kw 164
#9 MeSH descriptor Computing Methodologies explode all trees 4993
#10 (#1 OR #2 OR #3 OR #4 OR #5 OR #6 OR #7 OR #8 OR #9) 11246

#11 MeSH descriptor Data Collection explode tree 1 20640

#12 MeSH descriptor Database Management Systems, this term only 17

#13 MeSH descriptor Adverse Drug Reaction Reporting Systems, this term only 44

#14 MeSH descriptor Automatic Data Processing explode all trees 45

#15 case report form*:kw 77

#16 (diary OR diaries):kw 4

#17 crf*:kw 0

#18 (data NEAR/2 capture):kw 0

#19 data collection:kw 1536

#20 e-PRO:kw 0

#21 electronic NEAR/3 outcome*:kw 0

#22 (#11 OR #12 OR #13 OR #14 OR #15 OR #16 OR #17 OR #18 OR #19 OR #20 OR #21) 21720

#23 electronic data capture:kw 0

#24 edc:kw 0

#25 (clinical data management system* OR CDMS):kw 8

#26 clinical trial management software:kw 48

#27 (#23 OR #24 OR #25 OR #26) 55

Scopus search

Scopus, as of Nov 15, 2006

((TITLE-ABS-KEY-AUTH(rate* OR adopt* OR uptake OR up-take OR trend*)) AND ((TITLE-ABS-KEY(electronic data capture OR edc)) OR (TITLE-ABS-KEY(clinical data management system* OR cdms)) OR (TITLE-ABS-KEY(clinical trial management software)))) OR ((TITLE-ABS-KEY-AUTH(rate* OR adopt* OR uptake OR up-take OR trend*)) AND (TITLE-ABS-KEY-AUTH(e-pro OR electronic patient reported outcome*))) AND PUBYEAR AFT 1996

Google Scholar

as of Nov 15, 2006

internet clinical trials -PubMed

Related Articles Seed

Articles used as seeds for the related articles search:

1. Sahoo U, Bhatt A. Electronic data capture (EDC)--a new mantra for clinical trials. Qual Assur. 2003 Jun-Dec;10(3-4):117-21. PMID: 15764550
2. Velazquez I, Navarro X, Cobos A. [Electronic data capture. Impact on the quality of the clinical research] Med Clin (Barc). 2004;122 Suppl 1:11-5. Review. Spanish. PMID: 14980154
3. Marschner N. [Clinical investigations: innovative management of data. Electronic Data Capture: IOStudy Office] Zentralbl Gynakol. 2001 Aug;123(8):441-3. German. PMID: 11562806
4. Marks RG. Validating electronic source data in clinical trials. Control Clin Trials. 2004 Oct;25(5):437-46. PMID: 15465614

Figure 1: Search strategy for the systematic review.

Eligibility Criteria

Articles were included if they met the following criteria: the research method was a survey, had a focus on EDC technology, were published in English, contained original data, were not a press release, where the unit of analysis was a clinical trial and reported a percentage or a proportion (instead of a subjective scale, such as a Likert scale on frequency of use). Finally, reports on adoption within single pharmaceutical companies were excluded.

Two independent raters screened titles, abstracts, and keywords. If either of the ratings suggested the article was eligible then it was obtained for further assessment. The two raters calibrated their scores on a sample of 50 articles. If the Kappa inter-rater agreement between the two independent raters was lower than 0.7 then they reconciled their differences and performed a subsequent calibration until their agreement was higher than this threshold.

Selection Process

The selection process consisted of review of full-text articles that had appeared relevant by their title, abstract, or keywords. Eligibility criteria were applied to the full-text articles. Full text articles were reviewed independently by two reviewers. Disagreements were resolved by consensus. Figure 2 provides a modified QUOROM flow chart outlining the process for selecting identified EDC usage surveys.

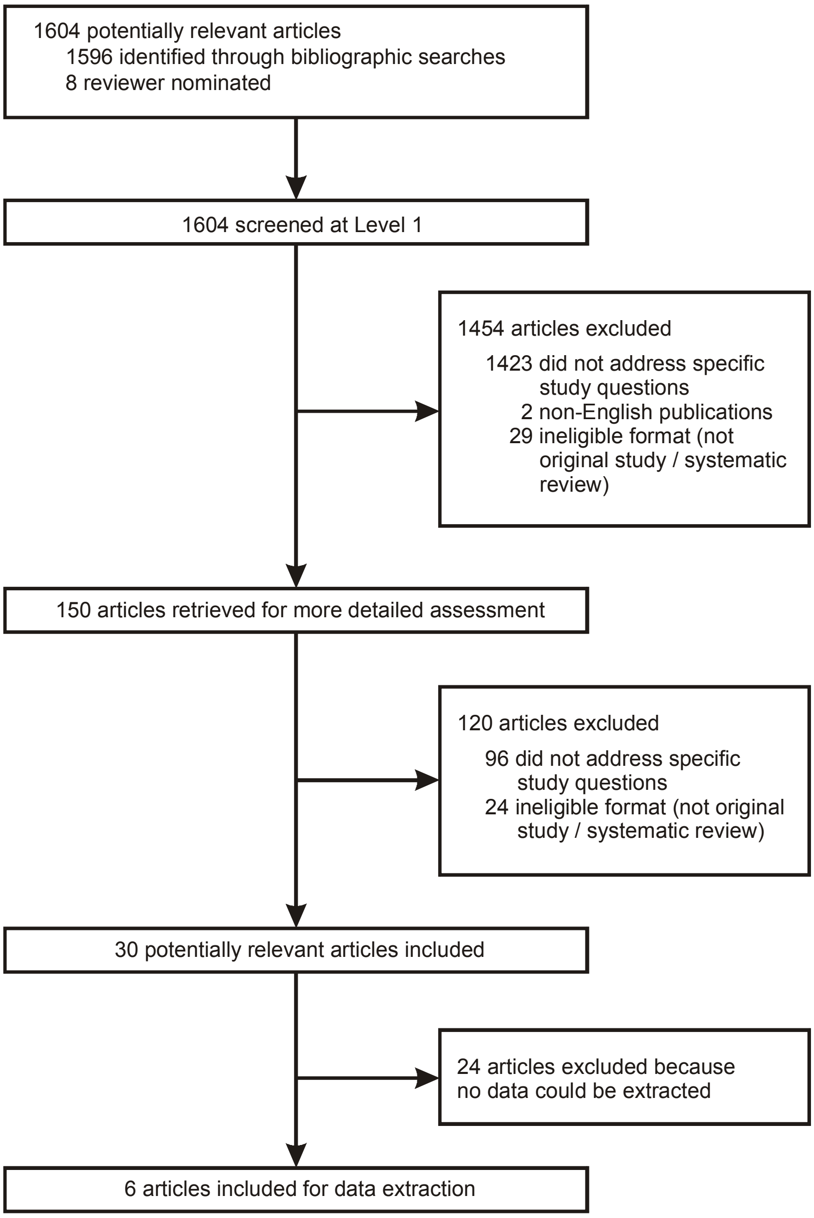

Figure 2: Modified QUOROM diagram for the systematic review.

Data Abstraction

Two reviewers abstracted the contents of each included survey. The proprietary SRS® system was used for screening and data abstraction.

Analysis

Quality assessment frameworks for descriptive study designs, such as surveys, were used to determine the methodological quality of the selected surveys and their reporting [3-7]. Each article was rated as high, medium, or low in terms of its quality by two independent raters based on the reporting of basic practices: use of a probability sampling method, reporting of item and unit non-response, analysis of non-response bias, the use of validated instruments, exact methods of analysis (e.g., whether the reported percentages are means or medians across organizations), and measures of variation or uncertainty around the adoption rates (such as confidence intervals).

A survey was judged to be of “high” quality if at most one practice above was not reported and adequate. A survey was judged of “low” quality if at least three of the practices above were not reported and/or not adequate. Surveys with two practices not reported or not adequate were judged to be “medium” quality.

Results

The body of evidence on the adoption rates of EDC is sparse. From a total of 1604 references that underwent an initial screening, only 30 were deemed potentially relevant. Full relevance assessment identified EDC adoption surveys that were reported upon in six articles [8-13] (see Table 1 for a summary).

None of these surveys was peer reviewed in a traditional academic sense, and much of their reporting lacked important information. Furthermore, for the studies using *web* surveys, reporting guidelines such as CHERRIES [7] were not followed. Therefore, the quality rating for all six surveys was *low*.

Table 2 shows the overall adoption rate over the five year period 2000-2004, and Table 3, Table 4, and Table 5 show more detailed adoption numbers by phase, type of organization (i.e., sponsor or CRO – Contract Research Organization), and geography.

Another survey identified a potential size effect with larger sponsors being more likely to use EDC for Phase I and post-approval studies (Phase IIIB/IV) [9]. For Phase III studies the difference among sponsor sizes is less pronounced [9].

The most recent data indicate that approximately one fifth of clinical trials use EDC systems (see Table 2), with more use reported in Phase III trials (see Table 5). In earlier years, CROs tended to be a higher adopter of EDC systems compared to sponsors, but they are now being overtaken by sponsors (see Table 3). However, the rate of increase for sponsors is greater, with the adoption among CROs somewhat stagnant over the same period (see Table 3). Adoption in North America has also been increasing since 2000, while it has shown a slight decline over time in Western Europe (see Table 4).

Year	Study Sponsors	Data Collection Mode	No. of Responses	Reports on Multiple Phases?	Stakeholder*			Geography*	
					CRO	Sponsor	Investigative Site	N. America	W. Europe
2000	ACRP [12]	Not specified	840 responses received	X	X	X	√	√	√
	CDISC & Centrewatch [8-11]	Not specified	Not specified	X	√	√	√	√	√
2001	Forrester Research [13]	Online questionnaire	400 responses received	X	√	√	√	X	X
2002	CDISC and CenterWatch [8-11]	Online questionnaire	750 questionnaires received	√	√	√	√	√	√
2003	CDISC & CenterWatch [9]	Not specified	Not specified	X	√	√	√	√	√
2004	CDISC and CenterWatch [9]	Online questionnaire	More than 380 responses received	X	√	√	√	√	√

ACRP: Association for Clinical Research Professionals
CDISC: Clinical Data Interchange Standards Consortium
CRO: Contract Research Organization
* In some cases the articles indicate that data was collected from a particular stakeholder or geography, but did not necessarily present the results stratified by these variables.
√: The information is reported; X: The information is not reported.

Table 1: Summary of studies that evaluate EDC adoption rate.

Year	Overall Adoption Rate
2000	12% [8, 9], 10% [12], 15% [10]
2001	37% used EDC on 10% to 20% of their trials, 11% on more than 20% of their trials [13]
2002	17% [9], 29% [10]
2003	18% [9]
2004	20% [9]

Table 2: Percentage of trials overall that are using EDC systems.

Year	Sponsors	Contract Research Organizations
2000	11% [8, 10, 11], 8%* [9]	16% [8, 10, 11], 11%* [9]
2002	24% [8, 10, 11], 18%* [9]	24% [8, 10, 11], 16%* [9]
2003	27%* [9]	8%* [9]
2004	30%* [9]	11%* [9]

Table 3: Percentage of trials using EDC systems across different organization types. These include the use of electronic patient reported outcome tools, such as patient diaries.

Year	Sponsors		Contract Research Organizations	
	N. America	W. Europe	N. America	W. Europe
2000	11%	7%	11%	13%
2002	24%	20%	24%	13%
2003	37%	4%	15%	3%
2004	39%	8%	19%	6%

Table 4: Percentage of trials using EDC across different organization types and region. These results include the use of electronic patient reported outcome tools, such as patient diaries [9].

Trial Phase	Sponsors	Contract Research Organizations	Investigative Sites
Phase I	30%	23%	8%
Phase II	30%	24%	26%
Phase III	34%	41%	52%
Phase IV	21%	33%	27%

Table 5: Adoption data from 2002 across phases and types of organizations. The percentages in the table refer to trials (data from [8-10]).

Discussion

It is clear that there is a paucity of good evidence on the adoption of EDC systems in clinical trials. Few organizations have conducted surveys on this topic, and these studies, at least based on what has been reported, are weak methodologically. The most recent study in 2004 indicates an adoption rate of 20%.

The best indication of trend comes from the CDISC surveys [9] with data from 2000, 2001, 2002, and 2004. The pattern seen is that of increasing use by sponsors, growing from 8% to 30% of trials, with no trend toward growth for CROs - the minor fluctuation in rates for this group are probably within the range of expected sampling variability. It should also be noted that the number of sponsors responding declined from the first to the last survey while the number of CROs reporting grew (data not shown) - changes in the response base over time may account for the trends seen.

References

1. Garritty C, El Emam K. *Who's using PDAs ? Estimates of PDA use by health care providers: A systematic review of surveys.* Journal of Medical Internet Research, 2006; 8(2):e7.
2. Jha A, Ferris T, Donelan K, DesRoches C, Shields A, Rosenbaum S, Blumenthal D. *How common are electronic health records in the United States ? A summary of the evidence.* Health Affairs, 2006; 24(6):w496-w507.
3. Gauthier B. *Assessing survey research: A principled approach (work-in-progress report)*. Proceedings of the Professional Marketing Research Society Annual Conference. 2001.
4. Council of American Survey Research Organizations. *CASRO guidelines for survey research quality*. 1998; Available from: [http://www.casro.org/]. Archived at: [http://www.webcitation.org/5FktyneTg].
5. *Standards and guidelines for statistical surveys*. 2006; Office of Management and Budget.
6. *Best practices for survey and public opinion research*. 2005; American Association for Public Opinion Research.
7. Eysenbach G. *Improving the quality of web surveys: The checklist for reporting results of internet e-surveys (CHERRIES).* Journal of Medical Internet Research, 2004; 6(3):e34.
8. Borfitz D. *Conspiring Forces Behind EDC Adoption.* CenterWatch, 2003; 10(2).
9. *Research project on attitudes, adoption and usage of data collection technologies and data interchange standards*. 2005; Clinical Data Interchange Standards Consortium (CDISC).
10. Kush R, Getz K, Whalen A, Lamberti M. *Attitudes, adoption and usage of technologies for e-clinical trials / EDC and data interchange standards*. 2002; CDISC-CenterWatch.
11. Korieth K, Zisson S. *CROs, EDC companies partnering for e-clinical trials adoption*. 2005; CenterWatch.
12. Prasad R. *Data Management Forum (presentation)*. Association of Clinical Research Professionals. 2002.
13. Barrett M, Brown E, Twist A. *Web Clinical Trials Break Through*. 2001; Forrester.
