# Supplementary material for: The Use of Electronic Data Capture Tools in Clinical Trials: Web-Survey of 259 Canadian Trials
Source: J Med Internet Res. 2009 Mar 9;11(1):e8. doi: 10.2196/jmir.1120 (PMC2762772; doi:10.2196/jmir.1120)
Supplement: Supplementary file 2 [file jmir_v11i1e8_app2.pdf]

# Multimedia Appendix 2: Questionnaire Development and Validation

In this appendix we describe the process that was used to develop the questionnaire for measuring EDC adoption, as well as the final questionnaire. To be precise, we defined a set of features that make up an EDC system. An electronic data capture and management system in use in a trial must have some subset of these features to be considered an EDC system.

1) Subjects are randomized automatically, either through an automated telephone response system or through a web interface
2) Subject visit data is entered through a web interface into electronic Case Report Forms (eCRFs)
3) The on-line system has standard dictionaries for coding adverse events
4) The on-line system has standard dictionaries for coding medicationsData validation happens automatically when data is entered into the eCRF (either right away or when you press the SUBMIT button) – for example, to check for out of range values
5) Data queries can be generated, printed, and tracked to closure
6) Subject recruitment can be tracked on-line for each/your site – for example, you can see a graph of recruited and not withdrawn subjects over time
7) The completion status of each eCRF for each subject can be tracked automatically on-line – for example, you can see which visits have complete data and which still have incomplete eCRFs for each subject
8) You can print empty or completed eCRFs to maintain hard copies at your site
9) The eCRFs can be 'locked' once they are validated to ensure that they cannot be modified any more
10) An audit trail is maintained for all data entry and modification
11) You can view the audit trail for all of the eCRFs for subjects that you are responsible for
12) You have a unique account and password to access the on-line system
13) The password expires after a certain period and needs to be reset
14) The system will automatically log you off after a period of inactivity
15) There is an automatic reminder for a subject's next visit
16) The system allows you to track medication inventory at the sites
17) The system provides automated support for generating invoices to the sponsor when certain milestones are met for each subject

**Figure 1:** Initial set of features constructed based on the literature.

## *Features of an EDC System*

To start off with, we defined the following types of features based on the literature and regulatory requirements [1-3]:

- Data collection: randomization, data entry coding.
- Validation: data validation, and query resolution.
- Tracking and monitoring: basic recruitment tracking.
- Regulation: meeting the basic requirements of 21 CFR Part 11 for security and audit trails.
- Project management: medication tracking and invoicing.

The initial set of features are shown in Figure 1.

To validate the feature set (i.e., ensure that the features in each group are complete, and to prioritize the features within each group) we conducted interviews with 13 experienced study coordinators and clinical trial data managers (henceforth *the experts*). The experts had been involved in a median of 6.5 clinical trials that used an EDC system and have been coordinating clinical trials for a median of 6 years.

The questions were written on 5"x1" slips of paper and arranged randomly on a table. The experts were asked to manually group the questions under the five headings mentioned above. Within each grouping they were asked if there are any other features that were missing, if any of the features were overlapping, and if any should be removed. Then they were asked to prioritize them within each group. The interviewees were also allowed to add new groups.

To determine whether the interviewees' grouping of features matches ours, we performed a numeric cluster analysis. The distance matrix for numeric cluster analysis is derived from the incidence matrix as follows: $d_{ij} = 1 - s_{ij}$ where $d_{ij}$ is the distance between features i and j. The similarity $s_{ij}$ is between features i and j. Similarity is defined as the proportion of interviewees who have placed features i and j in the same group. Miller [4] has shown that this is a metric distance matrix, and hence satisfies the criteria desirable for the application of numeric cluster analysis techniques [5]. We used the Ward linkage method [6] to cluster the groupings of the interviewees. The emergent five groups were: data entry, randomization, validation, tracking/monitoring, and regulations.

Denote the set of groups by $G = \{G_1, \ldots, G_q\}$ , $n$ as the number of interviewees, and $m$ as the total number of features that are being grouped and prioritized. Let $1 \le j \le m$ , $1 \le k \le m-1$ , $j \ne k$ , and $1 \le i \le n$ . Each group $G_r$ can be described as a set of features $G_r = \{g_{r,1}, \ldots, g_{r,m}\}$ . Let an interviewee's ranking be given by:

$$R_{rijk} = \begin{cases} 1 if\ g_{rj}\ is\, ranked > g_{rk}\ by\, interviewee\ i \\ 0\, otherwise \end{cases}$$

Then the global priority measure for each feature within a particular group is given by:

$$P_{rj} = \frac{\sum_{k} \sum_{i} R_{rijk}}{n(m-1)}$$

For each group we dropped any features receiving a priority less than 0.2. The final set of features are shown in the main text of the paper.

## *Piloting the Questionnaire*

The questionnaire resulting from the above process was then piloted by a different set 6 experienced study coordinators to ensure that it had a meaningful set of EDC features, was easy to understand and can be completed within a reasonable amount of time. Feedback from these coordinators was used to further improve wording and the layout of the questionnaire.

The final questionnaire is provided in Figure 2.

All of the following questions pertain to the clinical trial mentioned in your email invitation. Please answer all questions. Completion of this questionnaire should not take more than 5-10 minutes of your time. Thank you very much.

- Does your site use an electronic system / database for data collection and data management, either fully or partially, for this clinical trial (also known as a Remote Data Entry system or an Electronic Data Capture system) ? [Y/N]

**[if yes]**

Does the electronic system provide the following features:

1. Subject visit data is entered by sites through a web interface into electronic Case Report Forms (eCRFs) [Y/N/NA/DK]
2. The system provides a unique account and password to access the on-line system [Y/N/NA/DK]
3. The system provides an audit trail for all data entry and data modification [Y/N/NA/DK]
4. The system will automatically log you off after a period of inactivity [Y/N/NA/DK]
5. Subject recruitment can be tracked on-line for each/your site – for example, you can see a graph of recruited and not withdrawn subjects over time [Y/N/NA/DK]
6. The system allows you to track medication inventory at the sites [Y/N/NA/DK]
7. The completion status of each eCRF for each subject can be tracked automatically on-line – for example, you can see which visits have complete data and which still have incomplete eCRFs for each subject [Y/N/NA/DK]
8. Subjects are randomized automatically, either through an automated telephone response system or through a web interface [Y/N/NA/DK]
9. Data validation happens automatically when data is entered into the eCRF (either right away or when you press the SUBMIT button) – for example, to check for out of range values [Y/N/NA/DK]

**[if no]**

- Does your site use a fax based system to collect data and fax it to a central site for data entry during this clinical trial ? [Y/N]
- Is data collected in paper forms and sent to a central site by courier or mail for data entry? [Y/N]

**Figure 2:** Final questionnaire used in our survey.

# References

1. Paul J, Seib R, Prescott T. *The Internet and Clinical Trials: Background, Online Resources, Examples and Issues*. Journal of Medical Internet Research. 2005; Available from: [http://www.jmir.org/2005/1/e5]. Archived at: [http://www.webcitation.org/5REw579E0].
2. *The Waife & Associates EDC Report (v11)*. 2005; Waife & Associates Inc.
3. International Society for Pharmaceutical Engineering. *The Good Automated Manufacturing Practice (GAMP) Guide for Validation of Automated Systems in Pharmaceutical Manufacture*. 2002.

4. Miller G. *A psychological method to investigate verbal concepts.* Journal of Mathematical Psychology, 1969; 6:169-191.
5. Aldenderfer M, Blashfield R. *Cluster Analysis*. 1984: Sage Publications.
6. Everitt B. *Cluster Analysis*. 1993: Edward Arnold.
