# Supplementary material for: The Use of Electronic Data Capture Tools in Clinical Trials: Web-Survey of 259 Canadian Trials
Source: J Med Internet Res. 2009 Mar 9;11(1):e8. doi: 10.2196/jmir.1120 (PMC2762772; doi:10.2196/jmir.1120)
Supplement: Supplementary file 3 [file jmir_v11i1e8_app3.pdf]

Multimedia Appendix 3
CHERRIES Summary

Design	Survey Design	The unit of analysis was a Phase II/III/IV clinical trial with at least one site in Canada. These trials were identified from the clinical trials registries: *clinicaltrials.gov* and *www.controlled-trials.com* . Study coordinators at Canadian sites were contacted to answer the questionnaire.
IRB approval and informed consent process	IRB Approval	The study protocol was submitted to the REB at the CHEO Research Institute.
	Informed Consent	At the beginning of the survey form, the participants were informed that their responses would be used only for the purpose of this research and their identity or contact information would not be shared. They were told that filling out this survey would take approximately 5-10 minutes.
	Data Protection	A commercial web survey provider was used: SurveyMonkey. All communications with the respondents were encrypted. All data was hosted by SurveyMonkey. Neither sensitive nor personal information was being collected in this survey.
Development and pre-testing	Development and testing	We used a commercial service provider, so our testing was limited to checking that the on-line data collection forms have been designed and behave properly.
Recruitment process and the description of the sample having access to	Open vs. Closed	The survey was a closed survey. The survey tool automatically created e-tokens (long and complex URLs) that allowed access to the on-line survey form, and e-mailed them to the respondents. Each potential respondent received one token.
	Contact Mode	Respondents received e-mails explaining the goals and purposes of the survey and asked for their participation. E-mails included the tokens which took the potential

		respondents to the on-line survey form. After the first e-mail, three reminder e-mails were sent out at 1 week intervals.
	Advertising	No advertising was used.
**Survey Administration**	Web / E-mail	The contacts were made by e-mail. However, the survey was web-based. The respondents used their web-browsers to respond. The data was collected automatically after their submission.
	Context	Following the special URL (token) given in the e-mail, the respondents were only able to view the survey form. They were not shown any other content.
	Mandatory/ Voluntary	The respondents were able to view the survey form without filling out the survey and submitting their answers. Responding to the survey was voluntary. Upon clicking on the submission button on each page, it was checked whether the response was a complete response or not. The respondent was reminded and asked to answer in case any question was left unanswered.
	Incentives	Three iPod Shuffles were raffled to the respondents once the survey was closed.
	Time/Date	The survey was conducted between January and May 2008.
	Randomization	No items or questionnaires were randomized.
	Adaptive Questioning	Conditional questioning was used. Based on their answers to question 1, respondents were directed to answer either question set 2 or questions 3 and 4.
	Number of Items	The questionnaire is included in this article.
	Number of screens	The whole questionnaire totaled 4 on-line pages but due to skip logic, each respondent viewed only 3 on-line pages.
	Completeness check	Each submitted response was checked for completeness. This functionality was available in the survey instrument by making all of the questions mandatory.
	Review Step	Respondents were able to go back to previous pages in the survey and update existing responses until the survey was

		finished or until they have exited the survey. After the survey was finished, the respondents were not able to re-enter the survey.
**Response Rates**	Individual Response Rate	The response rate for those who were sent the survey was 78%. The response rate for those for whom we did have some trial contact information was 46%.
	Unique Site Visitor	Not applicable. Each potential respondent received a unique link to the survey, and only one response was allowed per link.
	View Rates	N/A
	Participation Rate	N/a
	Completion Rate	N/A
**Preventing multiple entries from the same individual**	Cookies used	Each respondent received a token which is a long and complex URL that can be used to complete the survey only once.
	IP check	Not used
	Log file analysis	No log file analysis was done.
	Registration	The user could view the survey page only until s/he submitted the completed survey. The survey was never shown again to this user with the token that s/he used.
**Analysis**	Handling of incomplete questionnaires	Each survey section had to be complete upon submission since the survey tool only accepted completed forms. However, with the conditional logic it was possible for respondents to abandon the survey midstream after completing a section, and this can result in incomplete surveys. There three abandoned surveys where only the first ("EDC Adoption") section was answered. These were treated as "Excel" trials (i.e., the EDC system had none of the nine features) to err on the conservative side.
	Questionnaires submitted with atypical time stamp	Time to fill out the survey was only tracked during the pilot study.
